# Computational and Experimental Analysis of Redundancy in the Central Metabolism of Geobacter sulfurreducens

**DOI:** 10.1371/journal.pcbi.0040036

**Published:** 2008-02-08

**Authors:** Daniel Segura, Radhakrishnan Mahadevan, Katy Juárez, Derek R Lovley

**Affiliations:** 1 Department of Microbiology, University of Massachusetts, Amherst, Massachusetts, United States of America; 2 Departamento de Microbiología Molecular, Instituto de Biotecnología, Universidad Nacional Autónoma de México, Cuernavaca, Morelos, México; 3 Department of Chemical Engineering and Applied Chemistry, University of Toronto, Toronto, Ontario, Canada; 4 Institute of Biomaterials and Biomedical Engineering, University of Toronto, Ontario, Canada; 5 Departamento de Ingeniería Celular y Biocatálisis, Instituto de Biotecnología, Universidad Nacional Autónoma de México, Cuernavaca, Morelos, México; Harvard University, United States of America

## Abstract

Previous model-based analysis of the metabolic network of Geobacter sulfurreducens suggested the existence of several redundant pathways. Here, we identified eight sets of redundant pathways that included redundancy for the assimilation of acetate, and for the conversion of pyruvate into acetyl-CoA. These equivalent pathways and two other sub-optimal pathways were studied using 5 single-gene deletion mutants in those pathways for the evaluation of the predictive capacity of the model. The growth phenotypes of these mutants were studied under 12 different conditions of electron donor and acceptor availability. The comparison of the model predictions with the resulting experimental phenotypes indicated that pyruvate ferredoxin oxidoreductase is the only activity able to convert pyruvate into acetyl-CoA. However, the results and the modeling showed that the two acetate activation pathways present are not only active, but needed due to the additional role of the acetyl-CoA transferase in the TCA cycle, probably reflecting the adaptation of these bacteria to acetate utilization. In other cases, the data reconciliation suggested additional capacity constraints that were confirmed with biochemical assays. The results demonstrate the need to experimentally verify the activity of key enzymes when developing in silico models of microbial physiology based on sequence-based reconstruction of metabolic networks.

## Author Summary

Geobacter sulfurreducens is a member of the *Geobacteraceae* family of micro-organisms that breathe metals and have a unique mode of metabolism. Stimulation of the activity of this species in the environment has been shown to result in the removal of radioactive contaminants in groundwater. Similarly, the respiration of these micro-organisms also has been linked to electricity generation in a microbial fuel cell. Both the rate of electricity generation and the efficiency of ground water clean-up can be enhanced through the improved understanding of the growth and metabolism of *Geobacteraceae*. In order to better understand the growth and metabolism of this organism, we had constructed a large-scale mathematical model of the metabolic network of this organism. Using this model, we identified reaction alternates that sustain metabolism in the event of gene deletions. We then experimentally confirmed the role of these metabolic reactions through gene deletion mutants and biochemical assays and improved the predictive ability of the mathematical model. Such an integrated computational and experimental approach can be used to study the activity and function of metabolic network in a rapid manner for other poorly characterized organisms of environmental relevance.

## Introduction

*Geobacter* species are of interest because of their natural role in carbon and mineral cycling, their ability to remediate organic and metal contaminants in the subsurface, and their capacity to harvest electricity from waste organic matter [1–3]. Geobacter sulfurreducens [4] is the most commonly investigated species of this genus because a genetic system [5], the complete genome sequence [6], whole genome microarrays [7] and genome-scale proteomics [8] are available. Furthermore, functional genomics studies have provided insight into the mechanisms of extracellular electron transport onto important electron acceptors such as Fe(III) oxides and electrodes [9–14].

G. sulfurreducens can use either acetate or hydrogen as the sole electron donors for Fe(III) reduction, and fumarate or malate can also be used as terminal electron acceptors [4]. An understanding of acetate metabolism in *Geobacter* species is required because acetate, secreted by fermenting organisms, is the dominant electron donor for *Geobacteraceae* in soils and sediments [15], and because recent studies have shown that the addition of acetate to uranium-contaminated aquifers can stimulate in situ bioremediation of uranium contamination by *Geobacter* species [16,17]. Previous studies have demonstrated that *Geobacter* species, and the closely related Desulfuromonas acetoxidans, oxidize acetate via the TCA cycle [18–20]. However, many other aspects of acetate metabolism, and central metabolism in general, are still poorly understood. To better understand the physiology of G. sulfurreducens, a constraint-based genome-scale metabolic model was constructed and used to investigate the unique physiology associated with the reduction of extracellular electron acceptors, such as Fe(III) [21]. The genome-scale model enabled the assessment of the impact of global proton balance during Fe(III) reduction on biomass and energy yields, and successfully predicted the lower biomass yields observed during the growth of a mutant in which the fumarate reductase had been deleted [22].

Furthermore, the network reconstruction revealed the existence of a number of redundant or alternate pathways in the central metabolism of G. sulfurreducens [21]. Recent genetic and in silico studies have shown that the presence of such redundant metabolic pathways, as well as isozymes, can enable metabolic networks to withstand genetic perturbations [23–26]. Experimental evidence for alternate optimal pathways have been observed in E. coli, where four metabolic gene deletion mutants had significantly different metabolic flux distributions, but similar overall growth rates [25]. Hence, the systematic investigation of the role of redundant pathways using in silico models can provide key insights into the properties of the metabolic networks.

Here we report on a coupled computational and experimental evaluation of potential redundant pathways during acetate metabolism in *G. sulfurreducens.* We demonstrate the need for redundancy in the acetate assimilation pathways, due to a coupling between the TCA cycle and acetate activation to acetyl-CoA, and also the inactivity of some of the predicted alternatives for pyruvate oxidation to acetyl-CoA. We also show that by using this information to constrain the model, its predictive capacity can be improved.

## Results/Discussion

### Identification of Redundant Metabolic Pathways

**Figure 1:**
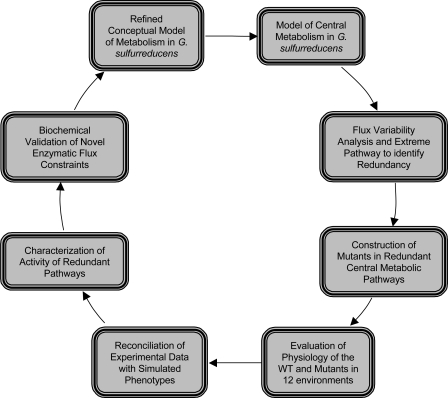
Schematic Describing the Experimental and Computational Approach for the Analysis of Redundant Pathways in Central Metabolism Involving Genetic, Physiological, Biochemical Methods and In Silico Modeling

**Figure 2:**
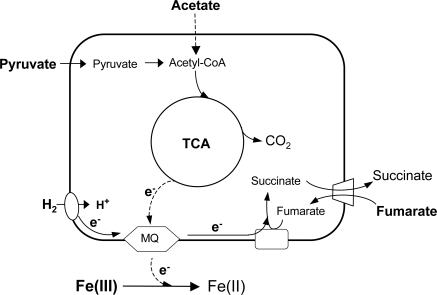
Metabolism of G. sulfurreducens with Respect to Possible Electron and Carbon Donors, and Electron Acceptors MQ, menaquinone pool.

A combined computational and experimental approach was used for characterizing key redundant metabolic pathways in G. sulfurreducens (see Figure 1). In order to identify all of the active redundant pathways in a specific environment, flux variability analysis (FVA) was used first to enumerate the reactions that participate in these pathways followed by Extreme Pathway Analysis (ExPA) to identify the alternate pathways. The FVA step is required as the direct application of the ExPA algorithm to a genome-scale network is computationally intractable [27]. This analysis was initially performed considering the use of acetate as electron donor and carbon source, with either fumarate or Fe(III) citrate as the electron acceptor (Figure 2).

**Figure 3:**
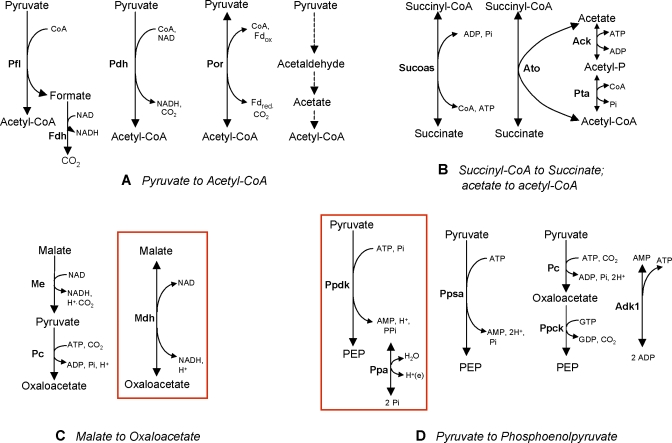
Optimal Equivalent Reactions Sets Studied The sets were identified in the metabolism of G. sulfurreducens using the FVA analysis during acetate oxidation with either fumarate or Fe(III) citrate as the acceptor, (A) pyruvate to acetyl-CoA and (B) succinyl-CoA to succinate; and non-optimal central metabolism alternate pathways studied, (C) the redundant pathways for conversion of malate to oxaloacetate and (D) the pathways for synthesis of phosphoenolpyruvate (PEP) from pyruvate. The energetically favorable pathways selected in the model simulations are enclosed in the red box. Ack, Acetate kinase; Adk1, Adenylate kinase; Ato, Acetyl CoA transferase; Fdh, Formate dehydrogenase; Me, Malic enzyme; Mdh, Malate dehydrogenase; Pc, Pyruvate carboxylase; Pdh, Pyruvate dehydrogenase; Pfl, Pyruvate formate lyase; Por, Pyruvate oxidoreductase; Ppa, diphosphatase; Ppck, Phosphoenolpyruvate carboxykinase; Ppdk, pyruvate phosphate dikinase; Ppsa, PEP synthase; Pta, Phosphotransacetylase; Sucoas, Succinyl-CoA synthetase.

FVA identified 32 reactions whose flux can vary with no effect on the growth rate and thus are predicted to participate in redundant pathways in the central metabolism of G. sulfurreducens (Table S2). From these 32 reactions, eight sets of redundant pathways that are comprised of reactions that are predicted to function as equivalent alternatives for optimal growth in G. sulfurreducens metabolism were identified with the Extreme Pathway Analysis algorithm [27]. These included reaction sets for the conversion of: pyruvate to acetyl-CoA, succinyl-CoA and acetate to succinate and acetyl-CoA glutamate to alphaketoglutarate, glutamate to alphaketoglutarate and glutamine, alphaketoglutarate to succinyl-CoA, AMP to ADP, and folate to tetrahydrofolate (see Figure 3 and Figure S1).

Some other reactions that could constitute alternatives providing redundancy, were not identified by this method because such reactions were considered inactive by the model when growing on acetate. One example is found in the malic enzyme that could participate in the conversion of malate to pyruvate (Figure S1), providing redundancy in the malate to pyruvate conversion, but it was not identified by the computational approach because this reaction is predicted to be inactive during optimal growth on acetate with either fumarate or Fe(III) citrate serving as the electron acceptor. However, this enzyme is predicted to be active under other conditions, such as during the absence of malate dehydrogenase activity (discussed in detail below).

### Genetic Analysis of Redundancy Predictions

Genetic and physiological analysis can help resolve the activities of the redundant pathways. To determine if the metabolic flexibility predicted by pathway analysis and modeling correctly described the physiology of G. sulfurreducens, and to further understand the role of some of the identified redundant pathways, genes encoding pyruvate ferredoxin oxidoreductase (Por, Figure 3A), phosphotransacetylase (Pta, Figure 3B) and acetyl-CoA transferase (Ato, Figure 3B), were inactivated (Table 1 and Figure S2). In addition to these reactions that are all stoichiometrically equivalent and are optimal alternatives, we also considered two cases of sub-optimal alternatives in the central metabolism for the evaluation of the predictive capacity of the model. These two pathways were identified by considering reactions which, when deleted in silico [21]*,* were predicted to result in sub-optimal growth relative to the wild type growth rate. One of these was the oxidative decarboxylation of malate by the malic enzyme, and the subsequent carboxylation of pyruvate, which could potentially substitute for the activity of the malate dehydrogenase (Figure 3C), but at the cost of an extra ATP. The other sub-optimal alternatives considered were the synthesis of PEP from pyruvate (Figure 3D), via the PEP dikinase (Ppdk, EC 2.7.9.1), the PEP synthase (Ppsa, EC 2.7.9.2), or the PEP carboxykinase (Ppck, EC 4.1.1.32)/pyruvate carboxylase (Pc, EC 6.4.1.1) pathway. Of the three pathways, the Ppdk pathway is energetically more favorable than either the Ppck or the Ppsa pathway, both of which are stoichiometrically equivalent. This is because the Ppdk pathway can lead to proton translocation via the diphosphatase reaction (Ppa) and thereby contributes to maintaining the proton gradient and ATP synthesis. For the analysis, we inactivated the genes encoding malate dehydrogenase (Mdh, Figure 3C) and PEP carboxykinase (Ppck, Figure 3D; Table 1).

**Table 1:**
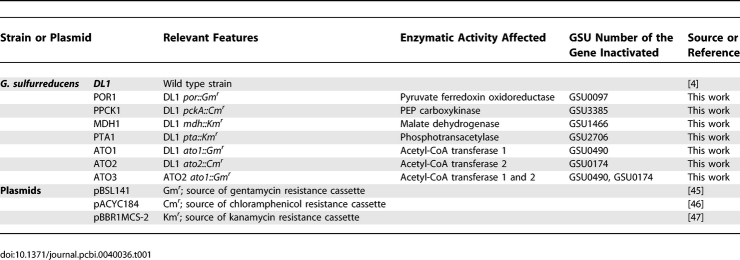
Bacterial Strains and Plasmids Used in This Study

The enzymes whose genes were inactivated were selected as they would provide additional information on the role of key central metabolic pathways (acetate activation, gluconeogenesis and anapleurotic pathways), which are conserved across different *Geobacteraceae,* and would enhance our understanding of the physiology of these acetate-utilizing bacteria.

Growth of the mutants with acetate as the donor, the condition used for redundant pathway identification, was evaluated. In addition, to further evaluate the predictive capacity of the model, the mutants were grown on combinations of acetate, pyruvate, and hydrogen as the carbon source/electron donor with either fumarate or Fe(III) citrate as the electron acceptor (Figure 2). This resulted in a total of 12 different growth conditions.

### Modeling of Wild Type Strain Phenotypes

**Figure 4:**
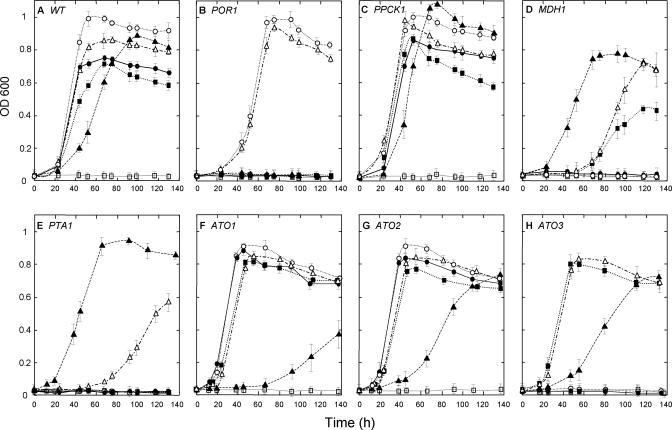
Growth Curves for Wild Type Strain (A) WT, (B) POR1, pyruvate ferredoxin oxidoreductase; (C) PPCK1, phosphoenolpyruvate carboxylase; (D) MDH1, malate dehydrogenase; (E) PTA1, phosphotransacetylase; (F) ATO1, acetyl-CoA transferase 1; (G) ATO2, acetyl-CoA transferase 2; (H) ATO3, acetyl-CoA transferase 1 and 2 mutant strains grown in medium containing fumarate as the electron acceptor, and acetate (•), acetate and hydrogen (), pyruvate** (**□**),** pyruvate and hydrogen (▴), acetate and pyruvate (**○**), or acetate, pyruvate, and hydrogen (**▵**) as electron donors. Growth was measured at A_600_ over time. Data are means of triplicates.

**Figure 5:**
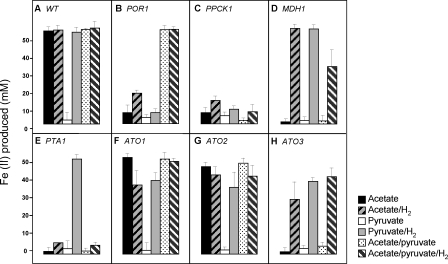
Fe(III) Citrate Reduction of Wild Type Strain (A) WT; (B) POR1, pyruvate ferredoxin oxidoreductase; (C) PPCK1, phosphoenolpyruvate carboxylase; (D) MDH1, malate dehydrogensae; (E) PTA1, phosphotransacetylase; (F) ATO2, acetyl-CoA transferase 1; (G) ATO2, acetyl-CoA transferase 2; (H) ATO3, acetyl-CoA transferase 1 and 2 mutant strains in Fe(III) citrate medium at 120 h using different electron donors. Log-phase cultures grown using fumarate as electron acceptor, and acetate (WT, PPCK1, ATO1, and ATO2 strains), acetate and hydrogen (MDH1 and ATO3 strains), and acetate and pyruvate (POR1 strain) as electron donors/carbon sources, were used as inoculum (3%). The data are the means for triplicate cultures.

The wild type strain could grow on all combinations of donors/acceptors except when pyruvate was the sole donor/carbon source with either fumarate or Fe(III) citrate as the acceptor (Figures 4A and 5A). However, G. sulfurreducens could grow in the presence of pyruvate and hydrogen with either acceptor. This indicated that pyruvate can be transported and used as a carbon source but it cannot serve as carbon and electron donor. The reason for this phenotype is not known and contrasted with predictions of the previously published model based on the presence of the transporter and enzymes needed for pyruvate oxidation [21]. Thus, it was necessary to incorporate an additional constraint on the pyruvate transport flux in order to ensure that pyruvate could contribute to growth as a carbon source but could not serve as the sole electron donor in silico. The pyruvate uptake constraint was chosen, as this constraint is active only during growth with pyruvate, and hence does not impact growth predictions in any other environment. The rate of pyruvate uptake was constrained to 0.15 mmol pyruvate/grams of dry weight per hour (gdwh), which is the rate required to meet the non-growth associated ATP maintenance demand (0.45 mmol ATP/gdwh) during growth with fumarate as the electron acceptor [21].

### Pyruvate Oxidation Redundancy

The pyruvate ferredoxin oxidoreductase (Por) reaction was evaluated because examination of the mutant phenotypes when growing on pyruvate as carbon source, would provide information about the functionality of the alternative reactions**,** pyruvate dehydrogenase (Pdh) and pyruvate formate lyase** (**Pfl), which could potentially substitute for pyruvate ferredoxin oxidoreductase activity, but with the production of different reduced electron carriers (Figure 3A). It is interesting to note that Por can be used not only for the anaerobic oxidation of pyruvate, but also for pyruvate synthesis [28], a role that likely occurs in this organism for the synthesis of three carbon compounds from acetate. In fact, previous in silico analysis has suggested that Por plays an important role in carbon fixation, converting acetate to pyruvate in G. sulfurreducens [21].

There were three putative Por encoded in the genome, two of them of the heterodimeric type, (gene clusters GSU1859–62 and GSU2052–54), and one of the homodimeric type (GSU0097 gene). The first two are more similar to indolpyruvate ferredoxin oxidoreductases, enzymes involved in the metabolism of aromatic amino acids (42% identity for AAM31789.1 from *Methanosarcina mazei* Go1, 42% identity for CAE09839 from Wolinella succinogenes DSM 1740). Furthermore, in both cases a gene coding for a phenylacetate CoA ligase, an enzyme involved in the metabolism of phenylalanine or in the aromatic catabolism of phenylacetic acid, is present immediately downstream, suggesting a putative role for the product of these genes in the reduction of a 2-oxoacid of the aromatic type. Therefore, the best Por candidate was GSU0097, encoding a putative homodimeric type enzyme [28]. It is similar to NifJ, a well characterized Por present in nitrogen fixing photosynthetic bacteria (59% identity to Q06879 from Nostoc sp. PCC 7120), where this enzyme has a role in providing electrons to ferredoxin or flavodoxin, the electron donors for nitrogenase [29].

The mutant deficient in GSU0097, designated POR1, lacked pyruvate-ferredoxin oxidoreductase activity (Table 2), indicating that the enzyme encoded by GSU0097 is the only functional pyruvate-ferredoxin oxidoreductase in G. sulfurreducens, under the conditions tested. Surprisingly, the POR1 strain was unable to grow with acetate as the carbon source and electron donor or with pyruvate as the carbon source and hydrogen as the electron donor (Figures 4B and 5B). The mutant did grow when both acetate and pyruvate were included in the medium. These results demonstrated that Por activity is the only way to produce sufficient pyruvate when growing on acetate. They also show that the predicted redundant pathways, pyruvate formate lyase, pyruvate dehydrogenase and another suboptimal alternative pathway through aldehyde dehydrogenase for the conversion of pyruvate to acetyl-CoA (Figure 3A and Table S3), are not functional, at least under the growth conditions evaluated. When cell extracts of the wild type strain were assayed (Table 2), we could not detect Pfl or Pdh activity, in accordance with the growth results. Therefore, the pyruvate formate lyase and pyruvate dehydrogenase reactions were inactivated in the model. In addition, the flux through the aldehyde dehydrogenase (Table S3) was constrained to be no greater than the corresponding value for the wild type case in order to limit the flux through this alternative pathway. New simulations with these additional constraints correctly predicted all the POR1 phenotypes (Table 3).

**Table 2:**
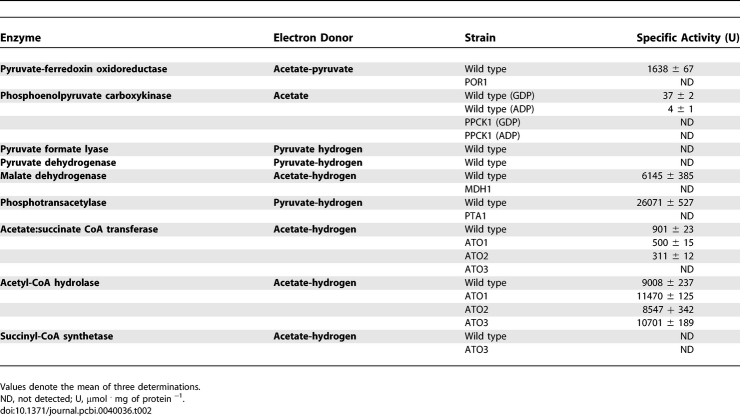
Specific Activities of Different Enzymes Measured in Cultures of the WT and Mutant Strains Grown Using Fumarate as the Electron Acceptor, and the Electron Donors Allowing the Best Growth of the Corresponding Mutant

**Table 3:**
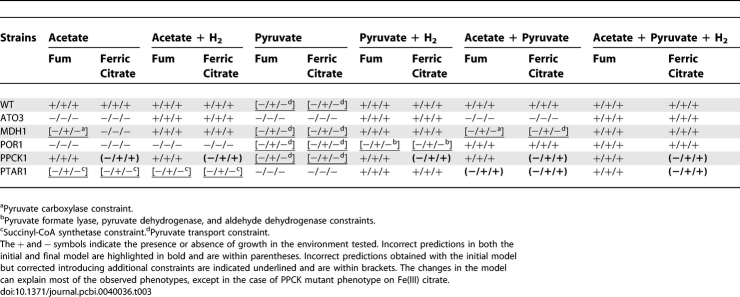
Comparison of the Experimental Growth Phenotypes with In silico Predictions from Two Cases: Those Utilizing the Model as Published in Mahadevan et al. (2006), and Those Obtained with the Additional Constraints Incorporated, Derived from the Analysis of the Experimental Data (In Vivo*/*In Silico*/*In Silico with Revised Constraints)

### Redundancy in the Synthesis of PEP from Pyruvate: Phosphoenolpyruvate (PEP) Carboxykinase Mutant

In the G. sulfurreducens metabolic network, gluconeogenic synthesis of phosphoenolpyruvate can occur through three possible ways with different energetic demands to provide the required PEP (Figure 3D). Hence, we evaluated the role of these pathways in gluconeogenesis through the analysis of a deletion mutant in phosphoenolpyruvate (PEP) carboxykinase (Ppck). The one gene in the G. sulfurreducens genome with clear homology to the Ppck enzymes from other organisms is GSU3385 (53% identical to BAD30010 from Corynebacterium glutamicum; [30]. The mutant in which GSU3385 was deleted, designated PPCK1, lacked the Ppck enzyme activity (Table 2), demonstrating that the GSU3385 gene codes for this enzyme. When the wild-type was assayed, activity was 10-fold higher with GDP than with ADP, suggesting that the enzyme belongs to the class of monomeric GTP dependent Ppck enzymes [31].

Strain PPCK1 grew at least as well as wild type with fumarate (Figure 4C) as the electron acceptor, but did not grow on Fe(III) citrate (Figure 5C). Growth on fumarate is consistent with model simulations which suggest that pyruvate phosphate dikinase (Ppdk) is the primary source for phosphoenolpyruvate (PEP) generation. The Ppdk reaction also produces diphosphate, which is hydrolyzed (Figure 3D) to translocate a proton across the cell membrane, resulting in an energetic advantage. The lack of growth on Fe(III) citrate was not predicted by the model. A possible reason for this is that Ppck activity could contribute to lower oxaloacetate levels, which is important because the conversion of malate to oxaloacetate by malate dehydrogenase is thermodynamically unfavorable (standard free energy change is + 29.7 kJ/mol [32]). A high oxaloacetate to malate ratio is not likely to occur when fumarate is the electron acceptor because malate pools are probably maintained at higher concentrations due to excess fumarate in the cell. Indeed growth on fumarate often results in secretion of malate [33]. Therefore, PPCK1 lethality when Fe(III) citrate is the electron acceptor is not because the phosphoenolpyruvate (PEP) requirements of the cells are not met by redundant pathways, but probably because of a disruption in oxaloacetate homeostasis. However, since metabolite concentrations are not represented in the Flux Balance Analysis metabolic model, oxaloacetate homeostasis constraint cannot be incorporated in the model.

### Redundancy in the Malate Oxidation

The inactivation of the Mdh reaction (EC 1.1.1.37, Figure 3C) was included in this analysis to further investigate the role of redundant pathways in the anaplerotic reactions connecting the TCA cycle to the glycolytic-gluconeogenic pathway. Deleting the one gene in the G. sulfurreducens genome with homology to malate dehydrogenase (GSU1466) eliminated malate dehydrogenase activity (Table 2). The malate dehydrogenase-deficient strain, designated MDH1, could grow with hydrogen as the electron donor, but not acetate (Figures 4D and 5D). Growth on hydrogen in the absence of malate dehydrogenase is expected, as it is consistent with other mutants with defects in TCA-cycle enzymes [22,34] because the electrons obtained from hydrogen are likely to flow directly to the menaquinone pool, avoiding the need for reducing equivalents derived from the TCA cycle to generate energy.

The lack of growth with acetate as the electron donor when Fe(III) citrate is the electron acceptor was predicted by the model. Although there is a predicted alternative pathway for conversion of malate to oxaloacetate involving the malic enzyme and pyruvate carboxylase (Figure 3C), this alternative pathway is not optimal because it consumes ATP in the pyruvate carboxylase step. Simulations predicted that this extra ATP cost would prevent growth with Fe(III) citrate as the electron acceptor because of the already low energy yields with Fe(III).

For growth on fumarate, the model predicted that, in order to compensate for the lack of Mdh, the flux through the pyruvate carboxylase would have to increase over 50 fold relative to the wild type flux distribution. The measured activity of the pyruvate carboxylase was low in the wild type, ca. 5 μmol/mg of protein/min. When pyruvate carboxylase flux is constrained at levels for the wild type cells, the model correctly predicts that MDH1 should not be able to grow with acetate as the carbon source, even with fumarate as the electron donor (Table 3).

### Acetate Activation Alternative Pathways and the Conversion of Succinyl-CoA to Succinate

In order to evaluate the potential redundancy in pathways for converting acetate to acetyl-CoA (Figure 3B), the gene coding for the phosphotransacetylase (GSU2706) was deleted. There was only one gene putatively coding for Pta in the G. sulfurreducens genome (72% identical to AP00550643 from Desulfuromonas acetoxidans DSM 684) whereas there were two genes whose products putatively code for Ack, GSU2707 and GSU3348 [6], so we decided to inactivate GSU2706. The GSU2706 mutant, designated, PTA1, lacked phosphotransacetylase activity whereas high activity was detected in the wild type (Table 2).

PTA1 was unable to grow with acetate as the electron donor (Figures 4E and 5E). This contrasted with the predictions of the model which indicated that acetyl-CoA transferase should be the primary provider of acetyl-CoA and that growth was possible on acetate and fumarate. Even the addition of pyruvate as a carbon source to meet the gluconeogenic carbon requirements did not rescue the mutant growth on acetate. PTA1 did grow when pyruvate and hydrogen were the carbon source and electron donor, respectively (Figures 4E and 5E). However, when acetate was supplied as the carbon source to cultures growing with pyruvate and hydrogen, growth on Fe(III) citrate was completely inhibited and growth on fumarate was partially inhibited (Figures 4E and 5E).

**Figure 6:**
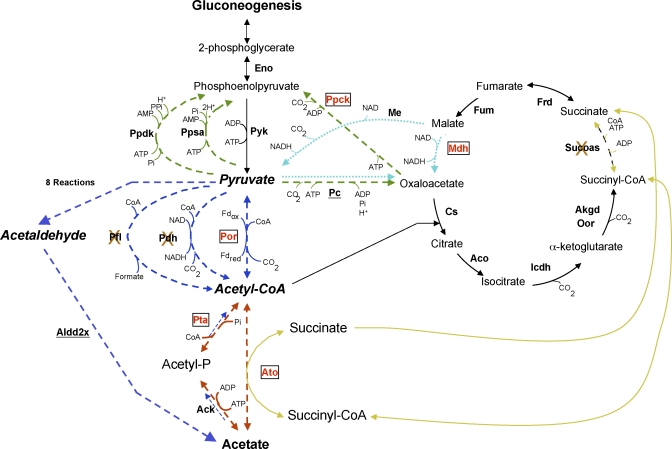
Central Metabolism in G. sulfurreducens Showing the Alternative Metabolic Pathways Studied (Dotted/Dashed Lines) The metabolic pathway in G. sulfurreducens after the refinement of the network based on the comparison of the in silico predictions with the physiological data from the mutant strains is shown. The underlined reactions represent cases for which the maximum allowed flux was constrained to the wild type levels. The boxes around the enzyme names highlight those proteins that were eliminated. The reactions with the “X” are constrained to have zero flux. Abbreviations are explained in Table S4.

In order to further evaluate this phenotype of PTA1, the predicted alternative pathway for succinyl-CoA synthesis catalyzed by succinyl-CoA synthetase was investigated. No succinyl-CoA synthetase activity was detected in cell extracts (Table 2), consistent with a previous report [33], even though genes coding for two putative subunits of this enzyme are present in the genome [6]. When the model was adjusted to remove the succinyl-CoA synthetase reaction it was found that for every acetyl-CoA molecule produced by acetyl-CoA transferase activity, one must be utilized in the citrate synthase reaction of the TCA cycle (Figure 6), effectively coupling the acetyl-CoA transferase flux with that of the TCA cycle. Thus, in order for acetate to be utilized for biomass production an alternative pathway for acetyl-CoA production is required. The acetate kinase/phosphotransacetylase pathway is apparently the only route for producing this acetyl-CoA. Growth is possible with hydrogen as the electron donor and pyruvate as the carbon source because pyruvate can be used for gluconeogenesis and hydrogen provides reducing equivalents.

The model simulations with an inactivated succinyl-CoA synthetase match the experimental observations in all the cases, except for growth in the presence of both acetate and pyruvate, or acetate, pyruvate and hydrogen. It may be that PTA1 accumulates acetyl phosphate, in a manner similar to mutants in other organisms that lack phosphotransacetylase [35]. Acetyl phosphate has been proposed to be a metabolic signal participating in the regulation of gene expression in other bacteria [36]. Therefore, it may be that acetate is affecting growth through a regulatory effect triggered by acetyl phosphate accumulation. This possibility is currently under investigation.

In order to further evaluate the mechanisms for formation of acetyl-CoA, the acetyl-CoA transferase activity (Figure 3B) was investigated. The model predicted that deleting the acetyl-CoA transferase activity would not permit growth on acetate, but that an acetyl-CoA transferase activity-deficient mutant should be able to grow with hydrogen as the electron donor. In the presence of hydrogen, the TCA cycle activity is not required for the generation of redox equivalents and consequently the acetyl-CoA transferase is predicted to be nonessential.

Two genes potentially encoding acetyl-CoA transferase enzymes were found in the G. sulfurreducens genome. These genes are also similar to well characterized acetyl-CoA hydrolases from S. cerevisiae (GSU0490 is 57% identical; [37,38]), and Neurospora crassa (GSU0174 is 58% identical [39]). The mutants lacking GSU0490 and GSU0174 were designated ATO1 and ATO2 respectively, and the double mutant in which both genes were inactivated was designated ATO3. Cell extracts of ATO1 and ATO2 had diminished acetyl-CoA transferase activity whereas the double mutant, ATO3, had no activity. The hydrolase activity was not affected in the mutants, demonstrating that GSU0490 and GSU0174 genes codes for acetyl-CoA transferases*.*

The single mutants ATO1 and ATO2 were able to grow under all the conditions tested (Figures 4F and 4G and 5F and 5G), as would be expected, because these mutants still have acetyl-CoA transferase activity. They did have a lower growth rate in the pyruvate-H_2_ medium than the wild type, an observation which could not be explained based on the Flux Balance Analysis model. ATO3, which completely lacked acetyl-CoA transferase activity, could only grow with hydrogen as the electron donor (Figures 4H and 5H). This follows the prediction of the model that growth on acetate is not possible because of the loss of the key step in succinate conversion in the TCA cycle. The ability of the mutant to grow with hydrogen as the electron donor and acetate as the carbon source indicates that the acetate kinase pathway for acetate activation is sufficient for this purpose.

### In Silico Model Refinement

The results demonstrate the continued need to experimentally verify the activity of key enzymes when developing in silico models of microbial physiology based on sequence-based reconstruction of metabolic networks. The initial version of the in silico model (Mahadevan et al., 2006) predicted the growth phenotype in 47 of the 72 growth conditions evaluated (Table 3). One of the most significant errors in the modeling was the prediction that pyruvate could serve as the sole electron donor to support growth. This prediction assumed that G. sulfurreducens had the necessary pyruvate transporters to support the required flux of pyruvate into the cell and the associated metabolic pathways to catabolize the assimilated pyruvate. The finding that G. sulfurreducens did not grow solely on pyruvate required that the pyruvate uptake be constrained to allow the use of pyruvate only as a carbon source (Materials and Methods). With this constraint the model accurately predicted the growth of the wild type for all conditions evaluated. However, there were 16 instances in which the model predicted the growth of mutants, because of predicted redundant pathways, when the mutants did not grow. These discrepancies between predictions and growth studies were helpful in identifying predicted pathways that are not actually functional or can not be considered truly redundant in G. sulfurreducens, at least under the growth conditions evaluated. These results have led to the following additional constraints in the model: the elimination of pyruvate formate lyase, pyruvate dehydrogenase, and succinyl-CoA synthetase reactions. Further constraints that limit the flux through pyruvate carboxylase, pyruvate transport and aldehyde dehydrogenase were also incorporated (see Figure 6). The model with the updated constraints was able to correctly predict 64 out of the 72 cases (89%) (Table 3). While these results are significant, it is important to emphasize that the model is essentially a reflection of our knowledge of metabolism and the improvement in the predictive capabilities of the model is the direct outcome of the characterization of the role of redundant pathways. Even with these additional constraints, the model could not predict the phenotypes in 5 conditions involving the PPCK (phosphoenolpyruvate carboxykinase) mutant (all during Fe(III) citrate reduction) and in 3 conditions involving the PTA (phosphotransacetylase) mutant, possibly due to accumulation of acetyl-phosphate, a regulatory feature, which can not yet be incorporated into the model due to lack of sufficient information.

In addition, an alternative approach for simulating the phenotype of knock-out mutations [40], in which minimization of the difference in flux distribution (MOMA) rather than growth maximization was the objective, was evaluated. However, applying MOMA with the additional constraints resulted in 14 incorrect predictions, of which 6 were false negatives. The remaining 8 were the same false positives predicted with the Flux Balance Analysis approach with the objective of growth maximization. The false negatives resulted from the fact that MOMA does not always identify non-zero growth solutions even if they are feasible, whereas such false negative predictions are less common in the Flux Balance Analysis approach.

In summary, the results demonstrate that the iterative comparison of the in silico and the in vivo phenotypes has led to additional information on the role and activity of the central metabolic pathways in G. sulfurreducens. Such integrated analysis of computational and experimental data can provide valuable insights on the activity and function of metabolic pathways in a rapid manner for poorly characterized organisms of environmental significance.

## Materials and Methods

### Model-based deletion analysis.

The previously described constraint-based in silico model of G. sulfurreducens [21] served as the basis for this analysis. Growth under different environments was simulated by modifying the constraints on the exchange fluxes of the corresponding growth medium constituents: electron donors such as hydrogen, pyruvate, and acetate, and electron acceptors such as Fe(III) citrate and fumarate. The presence of hydrogen in the environment was modeled by allowing a hydrogen influx of 10 mmol/gdwh. In the case of pyruvate, the in silico prediction that pyruvate could be utilized as the sole carbon and electron source if the pyruvate transporters are present, was inconsistent with physiological data, which indicated that G. sulfurreducens can grow on pyruvate only if an electron donor, such as hydrogen is also present (see Results). In order to ensure that pyruvate could contribute to growth as a carbon source but could not serve as the sole electron donor in silico, the rate of pyruvate uptake was constrained to 0.15 mmol pyruvate/gdwh, the rate required to meet the non growth associated ATP maintenance demand (0.45 mmol ATP/gdwh) during growth with fumarate as the electron acceptor [21]. The growth with acetate and fumarate was modeled by allowing the corresponding acetate and fumarate uptake rates to have flux values up to 5 mmol/gdwh and 25 mmol/gdwh respectively. Fe(III) citrate reduction in the presence of acetate was simulated by allowing acetate and Fe(III) uptake rates to have values up to 10 mmol/gdwh and 150 mmol/gdwh. These rates were chosen so that they were similar to experimentally observed values reported earlier [21,41]. Optimal growth rates with these constraints were calculated to be 0.055 hr^−1^ and 0.043 hr^−1^ for fumarate and Fe(III) citrate reduction, respectively, and were found to be consistent with experimental observations. If a particular substrate was not present in an environment, the uptake rate corresponding to the substrate was constrained to be zero.

Two different approaches to the prediction of a deletion mutant growth rate have been proposed in the past based on the assumptions of the cellular objective after a gene deletion [40,42,43]. Growth rates of in silico deletion mutants were calculated by these two approaches for all the mutants considered: (a) linear optimization which calculates the maximum possible growth rate for the mutant in the presence of a specific environmental condition [43] and (b) Minimization of Metabolic adjustment using the algorithm of Segre et al., 2002, which calculates the growth rate by using the optimal wild type flux distribution as a reference and minimizing the distance to the wild type solution (calculated by the Euclidean distance between the mutant and wild type flux distribution) in the flux coordinates. In silico deletion mutants with predicted growth rates lower than 0.001 hr^−1^ were considered to be lethal.

### Flux Variability Analysis (FVA) and redundant pathway identification.

The range of variation in fluxes for all various reactions in the model at the predicted optimal (maximum possible) growth rate was calculated with the previously described (Mahadevan et al., 2003) Flux Variability Analysis (FVA) algorithm. Briefly, this algorithm involves the maximization and minimization of every flux in the network subject to the stoichiometric constraints and an additional constraint that forces an optimal growth rate. The solution of this series of optimization problems results in the maximum and the minimum value of flux allowed for every reaction in the network, given the constraint that the growth rate is optimal [27]. Simulations were performed for growth with acetate as the electron donor and limiting substrate, and either fumarate or Fe(III) citrate serving as the electron acceptor. The acetate flux was assumed to be 5 mmol/gdwh for the case of fumarate reduction and 10 mmol/gdwh for the simulation of growth during Fe(III) citrate reduction so that the rates were representative of uptake rates observed experimentally [41]. In order to eliminate scaling issues in the FVA formulation, the upper bound and lower bound for fluxes that did not have any constraint was fixed to 1,000 and −1,000 mmol/gdwh respectively instead of –Infinity and Infinity. The fluxes that varied (range greater than 0.01 mmol/gdwh) were used to identify redundant pathways using a modified Extreme Pathway calculating algorithm [44] as previously described [27].

### Bacterial strain and culture conditions.

G. sulfurreducens strains used in this study (Table 1) were grown as previously described [5] in anaerobic pressure tubes with acetate (15 mM) as the electron donor and either Fe(III) citrate (56 mM) or fumarate (40 mM) as the electron acceptor. When indicated, 15 mM sodium pyruvate, hydrogen gas (0.59 atmospheres) or mixtures of these were used to replace or supplement the acetate. When hydrogen was used as electron donor, 10 ml hydrogen gas was injected into the headspace.

### Construction of mutant strains.

Mutations were introduced into the chromosome of G. sulfurreducens strain DL1 (ATCC 51573) [4] by homologous recombination. Construction of linear DNA fragments for gene disruption by recombinant PCR, electroporation and mutant isolation were performed as previously described [5].

Primers used for the construction of the various linear fragments utilized for gene disruption are listed and described in Table S1. The resulting genotypes of the various mutants constructed in this study are depicted in Figure S2. The plasmids used for generating the mutants are summarized in Table 1 [45–47]. All mutants were isolated in NBAFYE plates (NBAF medium amended with yeast extract) with the appropriate anoxic sterile antibiotic (200 μg/ml kanamycin, 20 μg/ml gentamycin, or 10 μg/ml chloramphenicol) as needed, and supplemented with 0.1% peptone, and 15 mM pyruvate to alleviate possible metabolic limitations generated by the gene inactivations. The plates were incubated in an anaerobic chamber under a 7% H_2_, 10% CO_2_, and 83% N_2_ atmosphere at 30 °C. A single isolate of each mutant was selected for detailed analysis and maintained with the adequate antibiotic. All the insertion-deletions were confirmed by PCR analysis.

In order to generate a mutant strain completely lacking the acetate:succinate Coenzyme-A transferase activity, a double mutant (ATO3) was constructed by electroporation of ATO2 mutant with the DNA fragment used to construct ATO1 (Supplementary Information: Figure S2).

### Analytical techniques.

Protein content was determined by a modification of the method of [48] using bovine serum albumin as protein standard. Growth of the cultures containing fumarate as electron acceptor was estimated by measuring turbidity at 600 nm. Fe(II) concentrations were determined with the ferrozine assay as previously described [49].

### DNA manipulations.

Genomic DNA was purified using the MasterPure Complete DNA & RNA purification kit (Epicentre Technologies) PCR product purification and gel extraction were carried out using the PCR purification kit and the Qiaquick gel extraction kit (Qiagen). Primers were purchased from Sigma-Genosys. All PCR amplifications were done using Taq DNA polymerase (Qiagen).

### Preparation of cell extracts and enzymatic assays.

Cell-free extracts were prepared from 100 ml mid-log cultures. Cells were harvested by centrifugation (12 min, 2450 × g rpm, 5 °C), washed with 50 mM potassium phosphate buffer, pH 7.3, containing 2.5 mM dithiothreitol, and centrifuged again. The cells were resuspended in 3 ml of the same buffer and were disrupted by sonication (20 times, 100 W, 10 s, in an ice water bath); the cell debris was removed by centrifugation (14,000 × g, 5 min, 5 °C), and the supernatant was further clarified by ultracentrifugation (125,000 × g, 1.5 h, 5 °C). The cell-free extracts used to determine pyruvate-ferredoxin oxidoreductase and pyruvate formate lyase activities were prepared under strict anoxic conditions using the same protocol except that the cells were disrupted in a French press at 40,000 kPa (two passages). All enzymatic assays were carried out at 30 °C. All specific activities are expressed in units per milligram of protein (1U = 1 μmol min^−1^).

Malate dehydrogenase activity (Mdh) was measured in the direction of oxaloacetate reduction [33] by monitoring the decrease of NADH absorption at 340 nm (E_340_ = 6.22 mM^−1^cm^−1^) in 1 ml assay mixtures containing 50 mM Tris-HCl, pH 8, 0.2 mM NADH, 2.5 mM oxaloacetate and cell extract.

Pyruvate carboxylase (Pc) and phosphoenolpyruvate carboxykinase (Ppck) activities were monitored at 340 nm in assays coupled to the NADH dependent reduction of oxaloacetate by malate dehydrogenase. The Pyc assay mixture (1 ml) contained 100 mM Tris-HCl pH 7.8, 5 mM MgCl_2_, 50 mM NaHCO_3_, 5 mM sodium pyruvate, 2 mM ATP, 0.1 mM NADH, 2 U porcine malate dehydrogenase (Sigma-Aldrich), and cell extract [50]. The Ppck activity assay mixture (1 ml) contained 100 mM Hepes buffer pH 7.8, 10 mM MgCl_2_, 0.5 mM MnCl_2_, 1 mM DTT, 50 mM NaHCO_3_, 0.25 mM NADH, 2.5 mM phosphoenolpyruvate, 2.5 mM GDP (or ADP), 10 U porcine malate dehydrogenase (Sigma-Aldrich), and cell extract [31].

Pyruvate-ferredoxin oxidoreductase (Por) activity was measured at 600 nm as the pyruvate-dependent reduction of methyl viologen (E_600_ = 12 mM^−1^cm^−1^) using a modified version of the method reported by [51]. The reaction mixture (1 ml) contained 50 mM Tris-HCl pH 7.5, 2.5 mM MgCl_2_, 1 mM thiamine pyrophosphate, 1 mM Coenzyme A (CoASH), 10 mM methyl viologen, and 10 mM sodium pyruvate. This assay was carried out in under anoxic conditions in sealed 1 ml cuvettes and all solutions were sparged with oxygen-free nitrogen.

Pyruvate formate lyase activity was determined at 340 nm using a coupled assay [52]. The reaction mixture contained 0.1 M Tris-HCl (pH 6.5), 0.2 mM CoA, 10 mM DTT, 1 mM NAD, 5 mM L-malate, 4 U of porcine citrate synthase (Sigma-Aldrich), 20 U of porcine malate dehydrogenase (Sigma-Aldrich), 50 mM pyruvate. The assay was carried out under anoxic conditions.

Pyruvate dehydrogenase was assayed by monitoring reduction of NAD at 340 nm [53]. The assay was carried out in 0.1M Tris-HCl (pH 6.5) containing 0.2mM of magnesium chloride, 0.01 mM calcium chloride, 0.3 mM thiamine pyrophosphate, 0.12 mM coenzyme A, 2.0 mM NAD, and 5 mM pyruvate.

Phosphotransacetylase (Pta) activity was measured in a coupled assay by monitoring reduction of NAD at 340 nm as previously described [54]. The reaction mixture contained 250 mM Tris-HCl (pH 7.8), 15 mM malic acid, 4.5 mM MgCl_2_, 2 mM CoASH, 22.5 mM NAD, 10 mM acetyl phosphate, 12 U of porcine malate dehydrogenase (Sigma-Aldrich), and 1.4 U of porcine citrate synthase (Sigma-Aldrich).

Acetate-succinate CoA transferase (Ato) activity was determined as described by Sohling and Gottschalk [55], via a coupled assay in which the product of the Ato reaction, acetyl-CoA, is condensed with oxaloacetate by the enzyme citrate synthase and the liberation of CoASH is monitored by measuring the reduction of 5,5′-dithio-bis(2-nitrobenzoic acid) (DTNB) at 412 nm (E_412_ = 13.6 mM^−1^cm^−1^). The reaction mixture contained 100 mM potassium phosphate buffer, pH 7.0, 200 mM, 1 mM oxaloacetate, 1 mM DTNB, 0.1 mM succinyl-CoA, and 3 U of porcine citrate synthase (Sigma-Aldrich).

Succinyl-CoA synthetase (Sucoas) was assayed in 50 mM Tris-HCl, pH 7.2, 100 mM KCl, 10 mM MgCl_2_, 0.4 mM ATP, 0.1 mM CoASH, and 20 mM sodium succinate [56]. Succinate-dependent succinyl-CoA formation was monitored at 235 nm (E_235_ = 4 mM^−1^cm^−1^).

Succinyl-CoA hydrolase (Sucoh) activity was measured by monitoring the liberation of free CoASH with DTNB at 412 nm [57]. The assay mixture (1 ml) contained 100 mM potassium phosphate buffer, pH 7.4, 0.125 mM DTNB, and 1 mM of acetyl-CoA.

### Supplementary information on flux variability analysis and extreme pathway analysis for identification of redundant pathways.

One approach to maintaining robustness of metabolism is through alternate pathways that can readily substitute in the event of loss of function. Metabolic modeling based on linear programming known as Flux Balance Analysis is effective in predicting large-scale growth phenotypes in different organisms. Flux Variability Analysis is an algorithm to identify those reactions that participate in alternate metabolic pathways. In this algorithm, in addition to the stoichiometric and capacity constraints, a growth rate constraint that forces the growth rate to be optimal is incorporated. Then, the flux through every reaction in the model is maximized and minimized in the formulation. If a reaction has an alternate pathway, then the flux through this reaction can be zero and yet optimal growth can be maintained as the flux gets rerouted through the alternate pathway. The range of flux is calculated as the difference between the maximum and the minimum possible value. Therefore, any reaction that has a non-zero range in the FVA problem has an alternate pathway. These reactions are compiled and augmented with the reactions in the reverse direction and used as the input to the Extreme Pathway Analysis algorithm. The redundant pathways are then identified from the list of predicted pathways. Further details are available in a previously published manuscript (Mahadevan and Schilling, 2003). However, if the number of reactions identified is small such as in the case of *G. sulfurreducens,* these pathways can be determined by manual inspection as shown in Table S2.

## Supporting Information

Figure S1 Additional Equivalent Reactions Sets Identified in the Metabolism of G. sulfurreducens Using the FVA Analysis During Acetate Oxidation with Either Fumarate or Fe(III) Citrate as the Acceptor (A,B) Glutamate to alphaketoglutarate.
(C) Glutamate to alphaketoglutarate and glutamine.
(D) Alphaketoglutarate to succinyl-CoA.
(E) AMP to ADP.
(F) Folate to tetrahydrofolate
(G) Malate to pyruvate, a set of reactions that are equivalent only if fumarate is assumed to be transported via a fumarate symport mechanism.
Adk1, Adenylate kinase; Adk2, Adenylate kinase (pyrophosphate); Akgd, Alphaketoglutarate dehydrogenase; Alad_L, L-alanine dehydrogenase; Alata_L, Alanine transaminase; Aspta1, Asparate transaminase; Aspt, Aspartase; DHFR, Folate reductase (2NADPH); DHFOR2, Folate reductase; DHFOR3, Dihydrofolate reductase; Fnor Ferredoxin: NADP oxidoreductase; Fum, Fumarase; Gludx, Glutamate dehydrogenase (NAD dependent); Gludy, Glutamate dehydrogenase (NADP dependent); Glusy, Glutamate synthase (NAD dependent); Glusz, glutamate synthase (ferredoxin dependent); Me, Malic enzyme; Mdh, Malate dehydrogenase; NADPH5, NADPH dehydrogenase, NADH5, NADH dehydrogenase; Oor, Oxaglutarate oxidoreductase; Ppck, Phosphoenolpyruvate carboxykinase; Ppik, polyphosphate kinase; Pyk, Pyruvate kinase. Other abbreviations are explained in Table S4.
(397 KB TIF)

Figure S2 Genotypes of the Various Mutants Constructed in This Study The structure of the gene clusters containing the inactivated genes, their transcriptional orientation, and the relative sizes of the deletions–insertions are shown. Black arrows represent the target genes and white blocks and arrows show the inserted cassettes. The *mdh* and *pta* genes were inactivated inserting a kanamycin resistance cassette in mutants MDH1 and PTA1 respectively; *por* and *ato1* were replaced with a gentamycin resistance cassette in mutants POR1 and ATO1, respectively; and a chloramphenicol cassette was used for the inactivation of *pckA* and *ato2* in mutants PPCK1 and ATO2, respectively. ATO3 mutant contained both *ato1::Gm^r^* and *ato2::Cm^r^* insertions. The transcriptional orientation of the inserted cassettes is the same as that of the target genes.
(160 KB TIF)

Table S1 Primers Used in the Recombinant PCR for the Gene Inactivations Described in This Study (1.6 MB TIF)

Table S2 The List of Reactions, the Range of Variation for Those Fluxes That Can Change without Effect in the Optimal Growth Rate, and the Equivalent Reaction Set Associated with the Reaction The list was calculated for acetate limiting growth with 1) acetate (5 mmol/gdwh) and fumarate and 2) acetate (10 mmol/gdwh) and Fe(III) citrate. Abbreviations are explained in Table S4.
(25 KB TIF)

Table S3 The Eleven Reactions That Are Predicted To Provide an Alternate Sub-Optimal Pathway for the Conversion of Pyruvate to Acetyl-CoA Abbreviations are explained in Table S4.
(20 KB TIF)

Table S4 List of Metabolite Abbreviations (53 KB TIF)

### Accession Numbers

The GenBank Database (http://www.ncbi.nlm.nih.gov/) accession numbers for the G. sulfurreducens proteins described in this report are as follows: Por (GSU0097), AAR33432.1; Ppck (GSU3385), AAR36775.1; Pta (GSU2706), AAR36078.1; Ato1 (GSU0490), AAR33822.1; Ato2 (GSU0174), AAR33509.1; and Mdh (GSU1466), AAR34840.1.
