# Correction: Computational and Experimental Analysis of Redundancy in the Central Metabolism of *Geobacter sulfurreducens*

**DOI:** 10.1371/annotation/67743d4d-2993-4d0c-951b-3f11ce65a8b4

**Published:** 2008-03-21

**Authors:** Daniel Segura, Radhakrishnan Mahadevan, Katy Juárez, Derek R Lovley

Under Funding, the grant number DE-FG02-01ER63221 was missing. The correct funding information reads: Funding. This research was supported by the Genomics:GTL program of the Office of Science (BER), US Department of Energy grants DE-FC02-02ER63446 and DE-FG02-01ER63221.

